# Role of Cytoskeletal Diaphanous-Related Formins in Hearing Loss

**DOI:** 10.3390/cells11111726

**Published:** 2022-05-24

**Authors:** Chiara Chiereghin, Michela Robusto, Valentina Massa, Pierangela Castorina, Umberto Ambrosetti, Rosanna Asselta, Giulia Soldà

**Affiliations:** 1 IRCCS Humanitas Research Hospital, Via Manzoni 56, 20089 Milan, Italy; chiara.chiereghin@fht.org (C.C.);rosanna.asselta@hunimed.eu (R.A.); 2 Experimental Therapeutics Program, IFOM ETS—The AIRC Institute of Molecular Oncology, Via Adamello 16, 20139 Milan, Italy; robusto.michela@gmail.com; 3 Dipartimento di Scienze della Salute, Università degli Studi di Milano, Via Di Rudinì 8, 20146 Milan, Italy; valentina.massa@unimi.it; 4 Casa di Cura Igea, Via Marcona 69, 20129 Milan, Italy; p.castorina@casadicuraigea.it; 5 Dipartimento di Scienze Cliniche e di Comunità, Università degli Studi di Milano and Fondazione IRCCS Cà Granda Ospedale Maggiore Policlinico, UO Audiologia, Via F. Sforza 35, 20122 Milan, Italy; umberto.ambrosetti@unimi.it; 6 Department of Biomedical Sciences, Humanitas University, Via Rita Levi Montalcini 4, 20072 Milan, Italy

**Keywords:** inherited sensorineural hearing loss, autosomal dominant auditory neuropathy, diaphanous-related formins, *DIAPH1*, *DIAPH2*, *DIAPH3*, mouse mutants

## Abstract

Hearing relies on the proper functioning of auditory hair cells and on actin-based cytoskeletal structures. Diaphanous-related formins (DRFs) are evolutionarily conserved cytoskeletal proteins that regulate the nucleation of linear unbranched actin filaments. They play key roles during metazoan development, and they seem particularly pivotal for the correct physiology of the reproductive and auditory systems. Indeed, in *Drosophila melanogaster*, a single diaphanous (dia) gene is present, and mutants show sterility and impaired response to sound. Vertebrates, instead, have three orthologs of the diaphanous gene: *DIAPH1*, *DIAPH2*, and *DIAPH3*. In humans, defects in *DIAPH1* and *DIAPH3* have been associated with different types of hearing loss. In particular, heterozygous mutations in *DIAPH1* are responsible for autosomal dominant deafness with or without thrombocytopenia (*DFNA1*, MIM #124900), whereas regulatory mutations inducing the overexpression of *DIAPH3* cause autosomal dominant auditory neuropathy 1 (*AUNA1*, MIM #609129). Here, we provide an overview of the expression and function of DRFs in normal hearing and deafness.

## 1. Introduction

The process of hearing relies on the proper functioning of auditory hair cells and on the integrity of all the cochlear structures contributing to mechanotransduction. In particular, the ability of hair cells to convert sound into nerve impulse is critically dependent upon actin-based cytoskeletal structures. Therefore, it should come as no surprise that mutations in genes encoding actin (e.g., *ACTG1*, encoding γ-actin, the predominant actin isoform in hair cells), actin-binding proteins (e.g., *TRIOBP*, an actin bundler, and *ESPN*, an actin crosslinker), actin-activated motor proteins such as myosins (e.g., *MYO1A*, *MYO3A*, *MYO6*, *MYO7A*, *MYO15A*, *MYH14*, and *MYH9*), and actin regulators (such as *DIAPH1* and *DIAPH3*) could cause hereditary deafness in humans [1].

This review summarizes the physiological role of a specific family of actin regulators, the Diaphanous-Related Formins, focusing on their expression and function in the auditory system. Data deriving from human families and genetically modified animal models are discussed. The cellular and molecular functions of DRFs have been extensively studied and are reviewed elsewhere [2,3]. An overview of the involvement of DRFs and other formin subfamilies in human diseases has also recently been published [4].

## 2. The Diaphanous Family

### 2.1. Evolution

Diaphanous-Related Formins (DRFs or Dia) are members of a metazoan subfamily of cytoskeletal regulators and constitute a major branch of the formin family, which comprises six other subfamilies in mammals: DAAM (disheveled-associated activator of morphogenesis), FRL (formin-related gene in leukocytes), FHOD (formin homology domain-containing protein), INF (inverted formin), FMN (formin), and delphilin [5,6]. They are involved both in actin nucleation and in the stabilization of microtubules [7].

The diaphanous (dia) gene was initially isolated as a gene involved in cytokinesis in the fruit fly, *Drosophila melanogaster*, during a screen for mutations causing male sterility [8]. It is located on chromosome 2L and is composed of five exons. It is considered an essential mitotic gene because null mutations cause lethality early on during pupal development.

**Figure 1:**
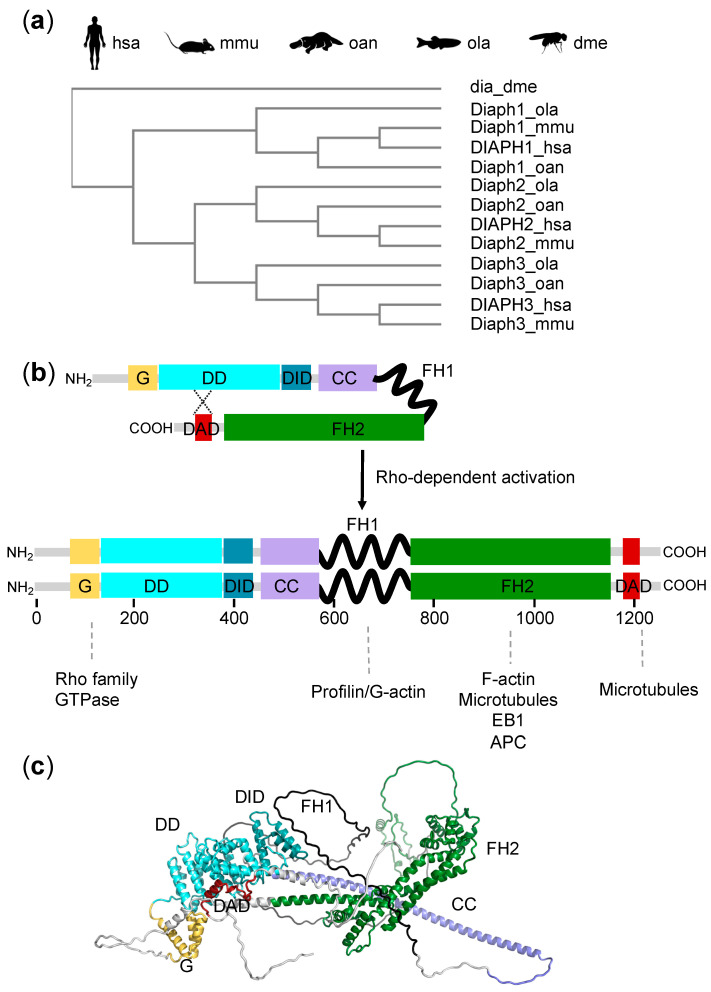
The Diaphanous-Related Formin (DRF) family. (**a**) Cladogram of Diaphanous-Related Formins in metazoa, based on the UniProt sequences and generated using Clustal Omega. The UniProt accession numbers of the selected sequences are: DIAPH1_hsa (O60610), DIAPH2_hsa (O60879), DIAPH3_hsa (Q9NSV4), Diaph1_mmu (O08808), Diaph2_mmu (O70566), Diaph3_mmu (Q9Z207), Diaph1_oan (A0A6I8NW07), Diaph2_oan (A0A6I8NX37), Diaph3_oan (F7CXN1), Diaph1_ola (H2LGS4), Diaph2_ola (A0A3B3I776), Diaph3_ola (A0A3P9MFT1), and dia_dme (P48608). hsa, *Homo sapiens*; mmu, *Mus musculus*; oan, *Ornithorhynchus anatinus* (platypus); ola, *Oryzias latipes* (medaka fish); dme, *Drosophila melanogaster* (fruit fly). (**b**) Schematic representation of the multi-domain structure of DRF proteins in the inactive autoinhibited form and in the Rho-activated dimeric form. Domains responsible for the interaction with actin and microtubules are indicated. CC: coiled coil domain; DAD: diaphanous autoregulatory domain; DD: dimerization domain; DID: diaphanous inhibitory domain; FH1: formin homology 1 domain; FH2: formin homology 2 domain; RBD: Rho Binding Domain. (**c**) Tridimensional structure of a diaphanous protein (monomeric), based on the AlphaFold prediction (AF-O08808-F1) for mouse Diaph1.

However, the evolutionary origin of the Dia formin family is probably older, as a protein with sequence similarities to metazoan Dia was identified in the choanoflagellate *Monosiga brevicollis* [9], and further phylogenetic analyses reinforced the idea that the emergence of Dia anticipated the evolution of animals and multicellularity [2]. In vertebrate genomes, the *dia* gene underwent duplication to give rise to three homologs called *Diaph1*, *Diaph2*, and *Diaph3* (Figure 1a). Gene duplicates are assumed to be completely redundant at the time of origin, and then they might get silenced by accumulating mutations (nonfunctionalization) or be maintained by natural selection. The latter might happen either in case of neofunctionalization, which is the acquisition of novel beneficial functions, or subfunctionalization, in which each gene copy retains only a part of the original function [10]. Concerning DRFs, nonredundant roles of mammalian Dia family members have recently been described in ciliogenesis and cilia maintenance, suggesting subfunctionalization. Indeed, the depletion of each of the three DIAPH proteins produced similar defects in ciliation and cilia length, which could be rescued only by the specific isoform. Moreover, the co-depletion of all DRFs did not show additive effects, indicating that the three proteins contributed independently, possibly acting at different steps of the ciliogenesis process [11,12]. Similarly, the nonredundant roles of the DIAPH1-3 proteins were described in cell motility and the capture of cortical microtubules. In this case, functional differences could be attributed to the differential binding of each DIAPH protein to a distinct set of interactors [13].

### 2.2. Structure

Members of the Dia family are multimodular proteins containing a series of domains and functional motifs (Figure 1b). They interact with numerous partners, including actin regulators, adapters, and signaling proteins. DRFs can be functionally divided into two halves. The C-terminal half is required for actin assembly and includes three structural and functional domains: (i) the profilin-binding FH1 (formin homology 1) domain, (ii) the actin-binding FH2 (formin homology 2) domain, and (iii) the DAD (diaphanous autoregulatory domain), which can interact with the N-terminal portion. The N-terminal half is the regulatory region of DRFs and contains the GTPase-binding domain (GBD) and the diaphanous inhibitory domain (DID) [14]. Furthermore, a dimerization domain (DD) and a coiled coil (CC) domain are present to mediate the dimerization of the N-terminal of DRFs, which are active as dimers [3]. The interaction between the N-terminal DID and the C-terminal DAD determines the transition of the DRF to its inactive conformation (Figure 1b). In addition, the first portion of the DID domain is also involved in the interaction with Rho family GTPases [15,16]. As a result of the partial overlap of the DAD and Rho binding sites, the binding of Rho GTPases induces the release of the DAD domain and stimulates actin nucleation functions (Figure 1b) [17]. The disruption of autoinhibition and/or Rho-dependent activation, by deletion or specific point mutation within the GBD, DID, or DAD domains, leads to a constitutively active protein with an abnormally upregulated actin polymerization [18,19,20].

Although the basic mechanism of Rho-dependent activation is shared among Dia family members, DIAPH1–3 show different specificity of interaction with small GTP-binding proteins: while Diaph1 specifically binds to and is activated by RhoA, B, and C, Diaph2 and 3 are more promiscuous and can also be activated by other Rho homologues such as Rac1, Cdc42, and RhoD. The structural basis of this specificity has been attributed to a small motif consisting of three asparagine residues (‘NNN’) in the mouse Diaph1, which is substituted for a Threonine-Serine-Histidine (‘TSH’ motif) in Diaph2 and 3 [5].

Crystal structures of N-terminal and C-terminal domains of the mouse Diaph1 protein as well as the structure of complexes between the N-terminal and C-terminal fragments have been resolved and have given insight into the mechanisms of actin nucleation, Rho-dependent activation, and autoinhibition [15,16,21,22,23]. No full-length crystals have been produced so far, mainly because the large unstructured FH1 domain is expected to prevent crystallization [22]. However, a prediction of the tertiary structure of the full-length Diaph1 polypeptide (accession number AF-O08808-F1) has become available through the AlphaFold machine learning approach (Figure 1c) [24,25]. Moreover, a three-dimensional reconstruction of the full-length mouse Diaph1 in the autoinhibited conformation was obtained by single particle electron microscopy at a resolution of 24 Å [26]. These and other structural and biochemical studies have shown that under basal conditions, diaphanous proteins form closed autoinhibited dimers in which actin filament binding is sterically obstructed. The efficient and full activation of diaphanous protein probably requires not only the binding of Rho proteins, but also additional factors such as membrane lipids, nucleation-promoting factors, and protein kinases [26].

### 2.3. Actin and Microtubule-Modulating Activity of Diaphanous Proteins

The role of diaphanous proteins in actin regulation is well-characterized. The FH2 domain alone is sufficient for actin nucleation by binding actin monomers and the barbed-end of filaments, while the elongation of existing actin filaments depends on the interaction of the FH1 domain with the G-actin-binding protein Profilin. Some DRFs, such as Diaph3 (but not Diaph1), also have actin filament side binding affinity, which allows actin bundling activity in addition to nucleation and elongation. Moreover, members of the diaphanous protein family interact with numerous actin regulators, adapters, and signaling components that can modulate their activity, either by promoting or inhibiting actin polymerization, or by controlling their localization within the cell and their association with the plasma membrane [2].

Besides actin polymerization, diaphanous proteins are known to contribute to the regulation of microtubule dynamics. Both Diaph1 and Diaph3 have been shown to bind microtubules directly, although with different stoichiometries. The interaction is mediated mainly by the FH2 domain for Diaph1, whereas for Diaph3, the C-terminal domain is also required for high-affinity binding [7]. All three DRFs mainly have a stabilizing effect on microtubules: the expression of constitutively active Diaph1 or Diaph3 promoted the formation of stable microtubules in several cell types, and Diaph2 was shown to be necessary for stable kinetochore–microtubule interaction [7]. The stabilizing activity of DRFs is partly intrinsic and partly due to their binding with other microtubule-interacting proteins such as EB1 and APC (adenomatous polyposis coli protein), which are both plus-end-tracking proteins involved in microtubule stabilization and positioning [7,27]. Because DRFs bind both actin and microtubules, and the interactions are mediated by the FH2 domain, the possibility that the binding of one cytoskeletal component could affect the binding of the other was also verified. Interestingly, the presence of actin monomers did not affect the binding of either Diaph1 or Diaph3 to microtubules; instead, actin polymerization was inhibited at various degrees in the presence of microtubules. In particular, the actin polymerization activity of Diaph1 was only partially blocked, whereas that of Diaph3 was completely inhibited [7].

Recently, diaphanous proteins were demonstrated to localize at the base of the cilia and to play significant and non-redundant roles in ciliogenesis and cilia maintenance, likely by mediating the trafficking of post-Golgi and recycling endosomal vesicles to the cilia [11,12]. More importantly, at least for DIAPH1, the effects observed are mediated by both its actin-dependent and microtubule-dependent activities [11]. However, the DIAPH3-mediated regulation of ciliogenesis and cilia length relies on microtubule but not actin function [12]. Worthy of note here is that both DRF depletion and basal body targeted overexpression produced cilia defects: the lack of any of *DIAPH1-3* genes caused a reduction in ciliation and cilia length, whereas their activation induced elongated cilia or cilia with a bulbous protrusion at their distal tips [11,12]. In addition, when different *DIAPH1* mutants were tested—some corresponding to likely gain-of-function mutations (e.g., NM_005219.4: c.3637C>T, p.R1213X),and others to loss-of-function mutations (e.g., NM_005219.4: c.2332C>T, Q778X and NM_005219.4: c.2099T>A, p.I530S)—all showed cilia abnormalities. None of the mutants were able to completely rescue the impairment of ciliogenesis caused by siRNA-mediated *DIAPH1* depletion [11].

Collectively, these observations provide new insight into the possible link between DRF function and hearing. First, even though diseases caused by defective cilia (collectively known as ciliopathies) typically show multiple organ disfunctions, some of them, such as the Bardet–Biedl syndrome, Alstrom syndrome, and Usher syndrome, are characterized by hearing dysfunction. Second, auditory hair cells possess specialized primary cilia known as kinocilia. Although kinocilia do not directly mediate auditory mechano-electrical transduction, they mediate hair cell morphogenesis and planar cell polarity, thus influencing the proper arrangement of stereocilia. Indeed, the loss of kinocilia results in abnormal and misoriented stereociliary bundles and auditory impairment. Such defects are seen, among others in mutants overexpressing *DIAPH3* [28,29], suggesting a possible link between the microtubule-modulating activity of the DIAPH3 protein, the (kino)cilia function, and hearing loss.

### 2.4. Expression of Diaphanous Proteins in the Inner Ear

In Drosophila, the dia gene is ubiquitously expressed, with a peak of expression observed within the first day of embryonic development, during late larval stages, and during early pupal stages [30]. Similarly, all Diaphanous genes are widely expressed, being detectable in most human tissues [31]. At the RNA level, *DIAPH3* shows lower and more restricted expression compared to *DIAPH1* and *DIAPH2*. At the protein level, DIAPH1 shows moderate to strong cytoplasmic reactivity in the immunohistochemistry analysis of the majority of tissues, with weaker staining in lymphoid tissues. DIAPH2 displays generally weak to moderate cytoplasmic staining, with the highest expression in the reproductive system (endometrium, fallopian tubes, and ovaries in females, testis in males), large intestines, and lung macrophages. Concerning DIAPH3, no immunohistochemistry data are currently available [31].

For a long time, relatively little information was present in the literature concerning the specific expression of diaphanous proteins in the inner ear, and the available data are limited to Diaph1 and Diaph3; the expression profile of Diaph2 in the auditory system is unknown. Diaph1 expression in the cochlea of wild-type adult mice (aged between 2 and 6 months) was first characterized in 2017 by both immunohistochemistry and immunofluorescence. In the Organ of Corti (OC), Diaph1 localizes to the inner pillar cells and at the base of the outer hair cells (likely within Deiters cell extensions). Moreover, Diaph1 is also found in spiral ganglion neurons (SGNs) and in the central part of the cochlear nerve [32]. A more recent study reported the endogenous expression of mouse Diaph1 during and after the differentiation of the OC, between P5 and P14 [33]. Besides confirming expression in various types of supporting cells, including Dieters cells and inner and outer pillar cells, Diaph1 was also detected in hair cells. During cochlear maturation, Diaph1 becomes progressively more expressed, with a basal-to-apical gradient, from a weak and regional distribution to a strong and widespread expression. Interestingly, Diaph1 shows specific enrichment at the apical junctional complexes (AJCs) between hair cells and supporting cells. These actin-based structures, which are composed of tight junctions and adherens junctions, have a fundamental role in maintaining the integrity and function of hair cells [33].

Diaph3 expression in the inner ear was assessed by cell-type-specific transcriptomic analysis in newborn and by qRT-PCR in adult (24 weeks of age) wild-type mice [28,34]. Low levels of expression were reported in all cell types, including sensory and non-sensory epithelium, blood vessels, neurons, and mesenchyme. Higher expression was detected in neurons as compared to sensory and non-sensory cells [34]. Unfortunately, it was not possible to determine the tissue and cellular distribution of the Diaph3 protein by immunohistochemistry because available antibodies showed nonspecific expression [28].

## 3. DRF Mutations and Hearing Loss

Mutations in *DIAPH1* and *DIAPH3* have been associated with different types of hearing loss (Table 1, Table S1). Alterations in all the other members of the formin family do not seem to cause an auditory phenotype, with the exception of inverted formin 2 (*INF2*) [35]. However, other types of genetic conditions, which do not include a hearing phenotype, have also been associated with mutations in the three diaphanous genes in humans (Table S2). For instance, biallelic mutations in *DIAPH1* cause seizures, cortical blindness, and microcephaly syndrome (*SCBMS*, MIM: #616632), while chromosomal rearrangements and copy number variation involving the *DIAPH2* gene have been recurrently found in patients with premature ovarian insufficiency (also known as *POF2A*, MIM: #300511), and *DIAPH3* mutations have been associated with autism spectrum disorders.

### 3.1. DIAPH1

**Figure 2:**
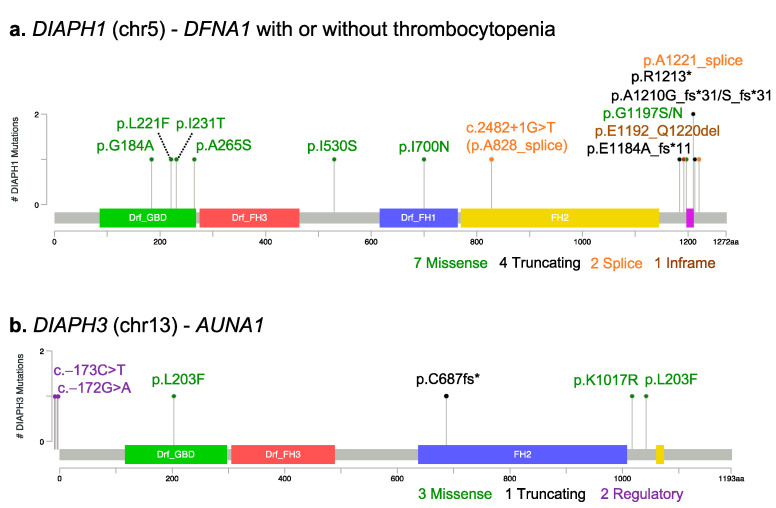
Mutations in DRFs and hearing loss. Lollipop plots showing genetic variants within human *DIAPH1* (**a**) and *DIAPH3* (**b**) that are associated with hearing phenotypes (see list in Table S1) and mapped on the corresponding protein. Mutations are color-coded based on the predicted functional effect (green, missense; black, truncating; orange, splice; brown, in-frame deletion/insertion; purple, regulatory). *, stop codon; fs, frame-shift. Plots were generated using Mutation Mapper, available at cBioPortal.

Heterozygous and likely gain-of-function mutations in the *DIAPH1* gene, located on chromosome 5, are associated with autosomal dominant deafness 1 with or without thrombocytopenia (*DFNA1*, MIM #124900) (Figure 2a and Table S1).

*DIAPH1* is one of the earliest mapped deafness genes. About 25 years ago, it was identified as the causative gene for *DFNA1* in a large Costa Rican family with early-onset (~10 years) low-frequency deafness, progressing to bilateral profound deafness in all frequencies. The causative variant (NM_005219.4: c.3661+1G>T) was a splicing variation that disrupted the donor splice site of intron 17. As a consequence, four bases are inserted into the mRNA, leading to a frameshift and the formation of a premature stop codon [37]. Since then, several families with autosomal dominant deafness have been reported to carry mutations in *DIAPH1*. The regions of the DIAPH1 protein that are more often mutated are the DAD domain at the C-terminal, and the GTPase-binding domain/DID domain at the N-terminal (Figure 2a), which are critical for the activation and regulation of the protein. Ueyama and colleagues demonstrated that a nonsense mutation (the NM_005219.4: c.3637C>T, p.R1213X), which caused the deletion of a portion of the DAD domain, disrupted the autoinhibitory DID–DAD interaction of DIAPH1, resulting in a mildly constitutively active protein [18]. Missense mutations in the GBD and DID domain of *DIAPH1* possibly act via a similar gain-of-function mechanism [20,38,39,40,41,42]. For instance, single-molecule fluorescence microscopy was recently used to confirm the pathogenicity of one of these mutation (p.A265S, located in the DID domain), suggesting that—in analogy with mutations in the DAD—it also disrupts the autoinhibitory interaction between the two domains [43].

Some patients who carry truncating variants (frameshift, nonsense, or in-frame deletion) in the DAD domain present with specific hematological symptoms in addition to deafness, such as thrombocytopenia, enlarged platelets, and increased bleeding tendency [32,39,44,45,46,47,48,49]. Interestingly, a relationship between the extent of the DAD truncation and the hematological phenotype has been suggested [47]. Recently, one heterozygous frameshift variant in the DAD domain has been reported in a Chinese family with auditory neuropathy, a specific type of hearing impairment characterized by the alteration of the auditory pathway but with a normal cochlear outer hair cell function [50].

Biallelic mutations in *DIAPH1* do not affect hearing function, but they have been causally linked with seizures, cortical blindness, and microcephaly syndrome (*SCBMS*, MIM #616632), a rare and severe autosomal recessive neurodevelopmental disorder (Table S2). In this case, the reported mutations are mainly frameshift or nonsense variants located in the FH2 domain, possibly leading to nonsense-mediated mRNA decay and thus resulting in the loss of *DIAPH1* function [51,52,53].

Studies in mice have contributed to a better understanding of the pathogenic mechanism and the genotype–phenotype correlation of the different types of *DIAPH1* mutations. For example, transgenic mice expressing the human NM_005219.4:c.3637C>T, p.R1213X variant showed progressive deafness caused by cochlear hair cell loss and stereocilia abnormalities. Conversely, *Diaph1* knock-out mice showed no hearing phenotype [19]. Altogether, these data further support the fact that *DFNA1* pathogenesis is due to the gain-of-function mutations in the *DIAPH1* gene. In addition, the observation of a myeloproliferative- and a myelodysplastic-like phenotype in a different *Diaph1* knock-out mouse [54] suggests that the *DIAPH1* gene plays a critical negative regulatory role in myelopoiesis and is consistent with the thrombocytopenia observed in humans carrying *DIAPH1* gain-of-function mutations. Conversely, *Diaph1*-deficient mice, despite demonstrating the crucial role of the gene in brain development, do not recapitulate the *SCBMS* phenotype observed in humans and present cerebral ventricular enlargement, but not microcephaly nor blindness [51]. Finally, *Diaph1* knock-out mice also show defects in T lymphocyte migration and proliferation [55,56], and this impaired function could explain the additional immunodeficiency observed in some *SCBMS* patients [53].

### 3.2. DIAPH2

The first studies associating *DIAPH2*, located on chromosome X, with a human phenotype reported the presence of chromosomal rearrangements (translocations, deletions, and duplications, listed in Table S2) involving this gene in patients with premature ovarian failure (*POF2A*, MIM: 300511), a dominant form of ovarian insufficiency characterized by secondary amenhorrea and premature menopause [57,58,59,60,61]. In some cases, ovarian dysgenesis is also present, which is consistent with the reported expression of mouse Diaph2 in the ovaries of developing mice [58] and the known role of the diaphanous protein in follicle cell division in female fruit flies [8]. However, no information on the presence of hearing impairment in *POF2A* patients with *DIAPH2* rearrangements are available. Hence, a possible link between pathogenic variants in the *DIAPH2* gene and deafness is still to be determined.

### 3.3. DIAPH3

Mutations in the human *DIAPH3* gene, which is located on chromosome 13, have been involved in nonsyndromic, autosomal dominant auditory neuropathy 1 (*AUNA1*, MIM #609129) (Figure 2b and Table S1). Auditory neuropathy is an inherited form of progressive human deafness characterized by abnormal or absent auditory brainstem responses but preserved cochlear outer hair cell function, as assessed by otoacustic emissions (OAE). The hearing impairment likely results from defects in the auditory nerve (VIII nerve), the inner hair cells, or the afferent synapses. The first *DIAPH3* mutation causing *AUNA1* was described in an American family of European descent [36,62]. The reported mutation was an heterozygous transition (NM_001042517.2:c.-172G>A) in a highly conserved element within the 5′ untranslated region of *DIAPH3*. The variant caused a 2–3-fold overexpression of *DIAPH3* mRNA and a 1.5-fold overexpression of DIAPH3 protein levels, consistent with a gain-of-function effect. Subsequently, a second Spanish family was reported to carry a different 5′UTR variant just one nucleotide upstream (NM_001042517.2:c.-173C>T), but no functional characterization was performed to confirm a gain-of-function effect [63]. In addition, missense variants in *DIAPH3* have been reported during genetic screening for mutations in individual patients with nonsyndromic sensorineural hearing loss or auditory neuropathy [64,65], but the actual pathogenicity of these variants is not clear. In the ClinVar database, apart from the well-characterized 5′UTR mutation, only one additional variant is classified as pathogenic (ClinVar Accession number VCV000984530): it is a single-base deletion that leads to a frameshift mutation, found at the heterozygous state in a patient affected by *AUNA1*.

Several animal models support *Diaph3* gain-of-function as being the cause of hearing loss in *AUNA1*. First of all, a drosophila mutant strain constitutively overexpressing the dia gene in the auditory organ showed a significant reduction in sound-evoked potential, mirroring the human *AUNA1* phenotype [62]. Similarly, at least two different transgenic mouse models overexpressing *Diaph3* have been generated, and both showed specific defects in the inner hair cells and progressive hearing impairment, with the preservation of the outer hair cell function, thus mirroring the human *AUNA1* phenotype. The defect seems to depend on the alteration of both the actin and the microtubule dynamics, with a profound alteration of the stereocilia structure and number [28,29]. Instead, loss-of-function *DIAPH3* mutants in both mice (knock-out model) and zebrafish (knock-down by antisense morpholino oligonucleotides) show an extremely severe phenotype, which in most cases leads to embryonic lethality. In zebrafish, lethality occurs at very early stages, before the formation of the auditory organ; in mice, although the majority of embryos died between E12.5 and E14.5, some animals survived until birth, showing severe developmental defects such as small brain, reduced body size, and facial deformities [66,67]. To better understand the function of *DIAPH3* in the brain, a conditional knock-out mouse was produced, in which the *Diaph3* gene was selectively inactivated in the cerebral cortex. These animals showed a profound alteration in neurogenesis, a marked reduction in the number of cortical progenitors and neurons, microcephaly, locomotor impairment, and defects in social interaction, which may indicate an autistic-like behavior [68]. This seems in line with clinical findings in humans, where *DIAPH3* was linked to autism in two independent studies (Table S2). The first case identified two different genetic defects in *DIAPH3* in the same patient: a missense substitution affecting the FH1 domain on one allele, and a large chromosomal deletion in the second allele [69]. The second case reported a de novo missense mutation within the *DIAPH3* FH2 domain together with another de novo mutation in a known autism-associated gene, *SET2* [70].

## 4. Conclusions

Despite extensive studies on the cellular and molecular functions of diaphanous-related formins, their role in the inner ear is still not well-understood, and the mechanisms by which specific genetic defects in the *DIAPH1* and *DIAPH3* genes primarily lead to a hearing phenotype is yet to be elucidated. For instance, the use of animal models has contributed to the characterization of the physiological expression of diaphanous proteins in the inner ear [32,33], and to the understanding of the gain-of-function effect on actin polymerization associated with a specific truncating mutation at the C-terminal of *DIAPH1* [19,33]. However, for other types of mutations, the mechanism by which DRF mutations lead to disease and affect or not the auditory system is less clear. In addition, in some cases, the animal model does not recapitulate the corresponding human phenotype.

Comprehensive genetic analysis and clinical characterization are available for several independent hearing-impaired families carrying mutations in the *DIAPH1* and *DIAPH3* genes. However, in some cases, a detailed clinical assessment of the affected patients and/or the functional characterization of the candidate pathogenic variants is lacking. The widespread use of whole-exome and whole-genome sequencing in the molecular diagnosis of inherited diseases, including hearing loss, is expected to provide evidence of novel and likely pathogenic variants in DRF genes. In particular, the development of sensitive and efficient molecular tests to verify the actual impact of the newly identified variants on the function of DRFs will help both in confirming pathogenicity and in understanding phenotype–genotype correlations. In this context, a novel and promising approach has recently been developed, which is based on the use of single-molecule fluorescence microscopy to evaluate the actin elongation activity of mutant DIAPH1 in cultured cells [43].

In conclusion, additional in vitro and in vivo studies and genetic analyses of human pedigrees will be needed to shed light on the redundant and non-redundant functions of DRFs in normal hearing and deafness.

## Figures and Tables

**Table 1:** Hearing loss associated with mutations in *DIAPH1* and *DIAPH3* genes.

	*DIAPH1*	*DIAPH3*
Inheritance	AD	AD
HL onset	First decade	Second decade ^1^
Affected frequencies	All	High frequencies more affected
Progression	Yes	Yes
ABR findings	Absent/abnormal	Absent/abnormal
Middle ear reflexes	Present	Absent
Affected structures	OHC	IHC, auditory nerve
Other symptoms	May be accompanied by thrombocytopenia	None

^1^ Earlier onset reported at 8 years of age [36]. AD: autosomal dominant; OHC: outer hair cells; IHC: inner hair cells.

## Data Availability

Not applicable.

## Supplementary Materials

The following are available online at https://www.mdpi.com/article/10.3390/cells11111726/s1, Table S1: Pathogenic mutations in DRF genes associated with a hearing phenotype; Table S2: Pathogenic mutations in DRF genes associated with diseases that do not include a hearing phenotype [71,72,73,74,75,76,77].
